# CpG-ODN induced antimicrobial immunity in neonatal chicks involves a substantial shift in serum metabolic profiles

**DOI:** 10.1038/s41598-021-88386-2

**Published:** 2021-04-27

**Authors:** Kalhari Bandara Goonewardene, Naama Karu, Khawaja Ashfaque Ahmed, Shelly Popowich, Betty Chow-Lockerbie, Lisanework E. Ayalew, Ruwani Karunarathna, Thushari Gunawardana, Mengying Liu, Suresh K. Tikoo, Marianna Foldvari, Philip Willson, Rupasri Mandal, David S. Wishart, Susantha Gomis

**Affiliations:** 1 Department of Veterinary Pathology, Western College of Veterinary Medicine, University of Saskatchewan, 52 Campus Drive, Saskatoon, SK S7N 5B4 Canada; 2 Department of Biological Sciences and Computing Science, University of Alberta, Edmonton, AB T6G 2E9 Canada; 3 Vaccinology and Immunotherapy, School of Public Health, University of Saskatchewan, Saskatoon, SK S7N 5E3 Canada; 4 School of Pharmacy, University of Waterloo, 200 University Avenue West, Waterloo, ON N2L 3G1 Canada; 5 Canadian Centre for Health and Safety in Agriculture, University of Saskatchewan, Saskatoon, SK S7N 5E5 Canada; 6 Analytical Biosciences and Metabolomics, Division of Systems Biomedicine and Pharmacology, Leiden Academic Centre for Drug Research, Leiden University, 2300RA Leiden, The Netherlands

**Keywords:** Metabolomics, Immunology, Antimicrobial responses, Immunotherapy, Innate immunity, Mucosal immunology, Microbiology, Antimicrobials, Bacteria

## Abstract

Synthetic CpG-ODNs can promote antimicrobial immunity in neonatal chicks by enriching immune compartments and activating immune cells. Activated immune cells undergo profound metabolic changes to meet cellular biosynthesis and energy demands and facilitate the signaling processes. We hypothesize that CpG-ODNs induced immune activation can change the host’s metabolic demands in neonatal chicks. Here, we used NMR-based metabolomics to explore the potential of immuno-metabolic interactions in the orchestration of CpG-ODN-induced antimicrobial immunity. We administered CpG-ODNs to day-old broiler chicks via intrapulmonary (IPL) and intramuscular (IM) routes. A negative control group was administered IPL distilled water (DW). In each group (n = 60), chicks (n = 40) were challenged with a lethal dose of Escherichia coli, two days post-CpG-ODN administration. CpG-ODN administered chicks had significantly higher survival (*P* < 0.05), significantly lower cumulative clinical scores (*P* < 0.05), and lower bacterial loads (*P* < 0.05) compared to the DW control group. In parallel experiments, we compared NMR-based serum metabolomic profiles in neonatal chicks (n = 20/group, 24 h post-treatment) treated with IM versus IPL CpG-ODNs or distilled water (DW) control. Serum metabolomics revealed that IM administration of CpG-ODN resulted in a highly significant and consistent decrease in amino acids, purines, betaine, choline, acetate, and a slight decrease in glucose. IPL CpG-ODN treatment resulted in a similar decrease in purines and choline but less extensive decrease in amino acids, a stronger decrease in acetate, and a considerable increase in 2-hydroxybutyrate, 3-hydroxybutyrate, formic acid and a mild increase in TCA cycle intermediates (all *P* < 0.05 after FDR adjustment). These perturbations in pathways associated with energy production, amino acid metabolism and nucleotide synthesis, most probably reflect increased uptake of nutrients to the cells, to support cell proliferation triggered by the innate immune response. Our study revealed for the first time that CpG-ODNs change the metabolomic landscape to establish antimicrobial immunity in neonatal chicks. The metabolites highlighted in the present study can help future targeted studies to better understand immunometabolic interactions and pinpoint the key molecules or pathways contributing to immunity.

## Introduction

Upon microbial entry, pathogens are sensed by host’s innate immune system through several pattern recognition receptors, predominantly toll-like receptors (TLRs)^1–5^. These receptors recognize pathogen-associated molecular patterns or molecules such as lipopeptides, lipoteichoic acid, flagellin, lipopolysaccharides, and unmethylated CpG motifs containing oligodeoxynucleotides (ODNs). This recognition leads to cell signaling cascades, which induce the secretion of pro-inflammatory cytokines such as interleukin (IL)-1β, IL-6, tumor necrosis factor as well as chemokines that attract phagocytic heterophils and macrophages to the site of infection^6,7^. This cascade ultimately leads to the development of adaptive immunity against the invading pathogens^2,8^. Chicken TLR21 and human TLR9 recognize CpG-ODNs containing GTCGTT motifs, and both have similar intracellular localization, signaling cascades, and cytokine induction patterns^9–14^. CpG-ODNs have great potential as immunotherapeutic agents and vaccine adjuvants against infections and cancer^15–18^. Several studies in humans^19–21^, mice^22^, cattle and sheep^23^, fish^24^, and chickens^25–27^ reported that CpG-ODNs initiate immune responses by activating immune cells and inducing cytokine secretion^16^. Our laboratory reported for the first time that standalone CpG-ODN treatment can protect against bacterial infections in chickens^28^. We showed that CpG-ODN administration protects chickens against Escherichia coli^2,26,29,30^ and Salmonella typhimurium infections^27^. Other studies have also demonstrated the antimicrobial function of CpG-ODN against Salmonella enteritidis infection^31,32^. We recently demonstrated that CpG-ODN treatment accelerates immune development by enriching immunological niches in chicks^2,33^. Furthermore, our recent data established a cause-and-effect relationship by showing that the levels of CpG-ODN-induced immune enrichment strongly correlate with the levels of protection against *E. coli* infection^34^. Regardless of recent advances, further investigations are needed to understand the mechanisms of CpG-ODN induced antimicrobial immunity better.

Several recent studies in humans and mice have suggested that energy metabolism significantly regulates immune cell fate and functions^35–37^. Macrophages and dendritic cells (the sentinel cells) were shown to have increased glucose metabolism^38^ and increased expression of the glycolytic enzymes, glucose-6-phosphate dehydrogenase and hexokinase^39^. Naïve resting T lymphocytes utilize oxidative phosphorylation for ATP generation, whereas aerobic glycolysis and glutaminolysis are the main methods of energy generation in activated T lymphocytes^40–43^. It was recently reported that the activation of TLR4 by bacterial lipopolysaccharides in neutrophils increases glucose consumption^44^. Cells stimulated via pattern recognition receptors (PRR) and pathogen-associated molecular pattern (PAMP) interactions undergo profound metabolic changes, which is important not only for the signaling processes but also for biosynthesis and energy production^45^.

Metabolomics offers an excellent route for exploring the molecular connections between immunity and metabolism. In particular, nuclear magnetic resonance (NMR) spectroscopy and mass spectrometry (MS) can be applied to identify metabolites (the end products of biological responses) in biofluids to better understand disease-induced and immunity induced processes and responses. Despite the potential involvement of the metabolic changes in shaping the immune responses, to the best of our knowledge, no extensive metabolomic profiling for CpG-ODN-induced immunity has been carried out.

In the present study, we hypothesized that CpG-ODNs potentially regulate metabolic pathways that control the development of antimicrobial immunity. Antimicrobial immunity, as induced by intramuscular (IM) administration of CpG-ODN, has been the gold standard to assess challenges against several bacterial pathogens^26–28,30^. We recently found that intrapulmonary (IPL) delivery of CpG-ODN also induces antimicrobial immunity in a dose-dependent manner^29,34^. Therefore, in this study, we compared NMR-based serum metabolomic profiles in chickens treated with IM versus IPL CpG-ODNs or distilled water (DW) control.

## Results

### Immunoprotective efficacy of intramuscular and intrapulmonary delivery of CpG-ODN compared to distilled water control group against *E. coli * septicemia

**Figure 1:**
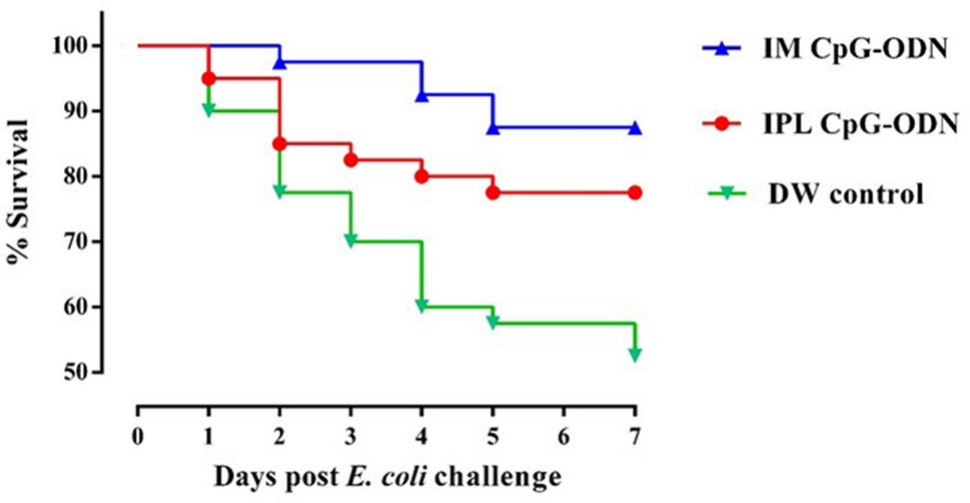
Survival percentages of the birds following lethal *E. coli* infection. Day-old neonatal chicks were treated with 50 µg of CpG-ODN intramuscularly (IM) or mucosal delivery via intrapulmonary (IPL) route [6 mg CpG-ODN aerosolized in a closed 0.036 m^3^ acrylic chamber containing 60 birds for 30 min] or treated with aerosolized distilled water (DW) as control. On the second-day post-treatment, the birds in each group (n = 40) were challenged with 1 × 10^5^ CFU or 1 × 10^6^ CFU of *E. coli* per bird, subcutaneously in the neck. The mortality was recorded until seven days post-challenge. Birds that received IM CpG-ODN (blue) and IPL CpG-ODN (red) treatments showed significantly better survival than the DW control (green) group (*P* < 0.05) over seven days post challenge.

**Figure 2:**
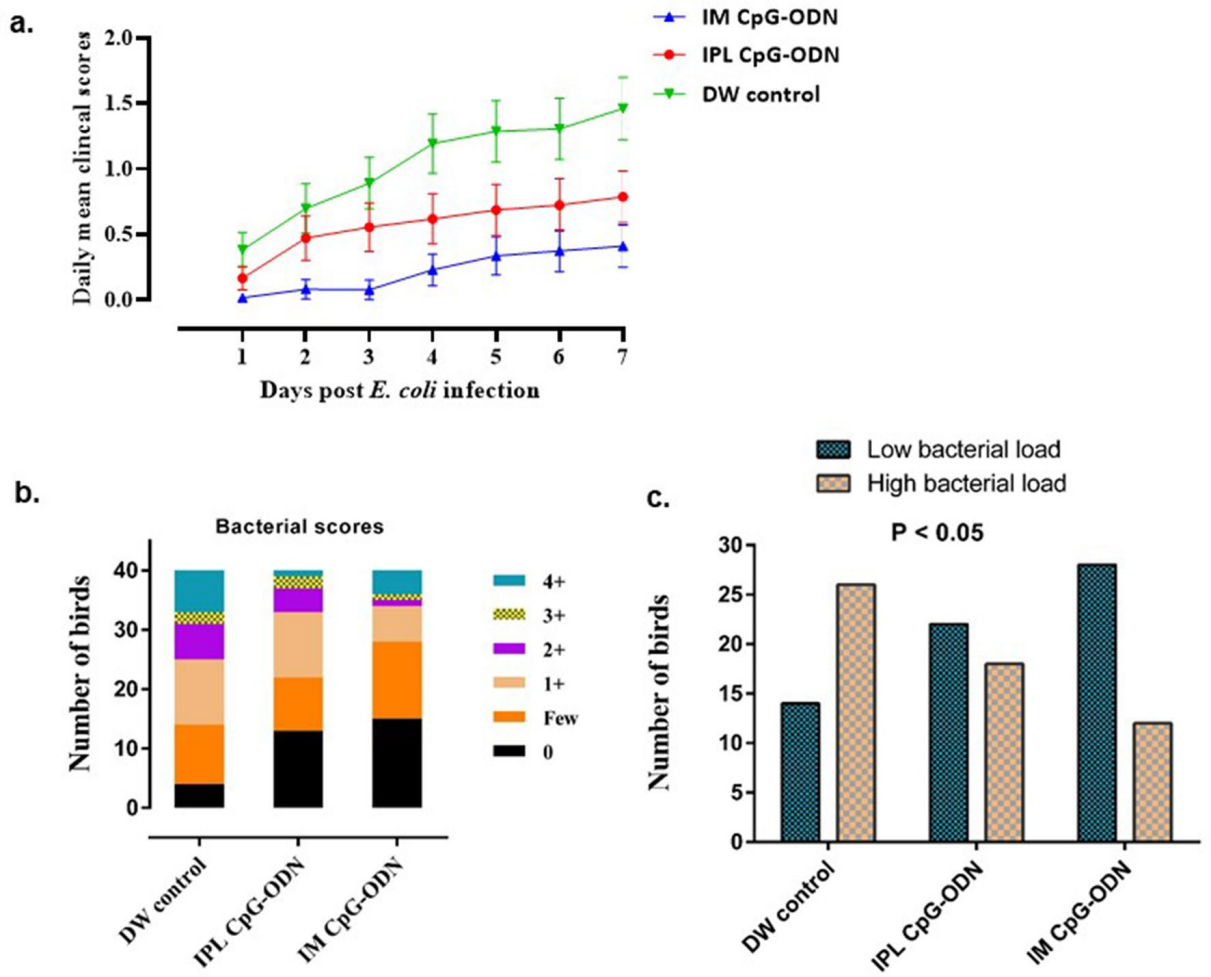
Cumulative clinical scores and air sacs bacterial loads in neonatal chicks following *E. coli* challenge. Mean cumulative clinical scores (CCS) of each group (**a**), various scores of bacterial growth isolated (**b**), and birds with low and high bacterial loads (**c**) in neonatal chicks (n = 40/group) following *E. coli* challenge. (**a**) Each data point in line represents the mean CCS value of individual groups on each day, and vertical lines indicate standard error of the mean. The mean CCS values of the IM CpG-ODN group (blue line) and the IPL CpG-ODN group (red line) were significantly lower compared to the IPL DW control (green line) group (*P* < 0.001). (**b**) Bar graph shows bacterial scores (0, few, 1 + , 2 + , 3 + & 4 +) of swabs^2^ taken from air sacs of birds and cultured on 5% Columbia sheep blood agar by the quadrant streaking method. Higher the bacterial scores greater are the bacterial loads. Increased bacterial load was observed more frequently in lesions from birds in the DW control than in the CpG-ODN groups. (**c**) For the statistical analysis (chi square test) on the bacterial scores as indicated in Fig. 2b, birds in each group were divided into two categories representing the low bacterial loads (no growth & few colonies) and high bacterial loads (1 + , 2 + , 3 + & 4 +). Bar graph shows the number of birds in each group with low and high bacterial loads. The IM CpG-ODN and IPL CpG-ODN administered groups displayed significantly (x^2^ = 9.911, *P* = 0.007) lower bacterial load compared to the DW control group.

Chicks that received CpG-ODN either through the IM or IPL routes or DW controls were challenged with lethal doses (1 × 10^5^ or 1 × 10^6^ CFU) of a pathogenic strain of *E. coli *. During the seven days of challenge experiments, chicks that received CpG-ODN were significantly protected compared to saline controls (Fig. 1). We found that CpG-ODN delivery through the IM route induced a higher survival against bacterial challenge compared to IPL CpG-ODN delivery. We also calculated the daily mean CCS for each chick through the seven-day observation period after E. coli challenge. The birds that received CpG-ODN IM or IPL had significantly lower CCS values (*P* < 0.001) compared to the DW controls (Fig. 2a); the lowest CCS values were in birds that received IM CpG-ODN. To measure the bacterial loads in different groups, we used a semi-quantitative estimate of E. coli isolation on Columbia sheep blood agar by the quadrant streaking method. In this method, clockwise streaking is done on the agar plate, and bacterial thinning occurs as streaking goes from quadrant 1 to quadrant 4. Therefore, the isolation of bacterial colonies in the higher quadrant suggests higher bacterial load in the sample. The number of birds with various bacterial loads is shown in Fig. 2b. These data clearly indicate CpG-ODN treatment led to a substantial reduction in bacterial loads. When birds in each group were divided into low and high bacterial load categories as mentioned in the materials and methods section, we found that the CpG-ODN administered birds had a statistically lower bacterial load in contrast to the DW controls; χ^2^ = 9.911, *P* = 0.007 (Fig. 2c). Birds that succumbed to challenge or were euthanatized had lesions such as pericarditis, airsacculitis or combination of airsacculitis together with pericarditis or polyserositis.

### Effects of CpG-ODNs on serum metabolites

**Figure 3:**
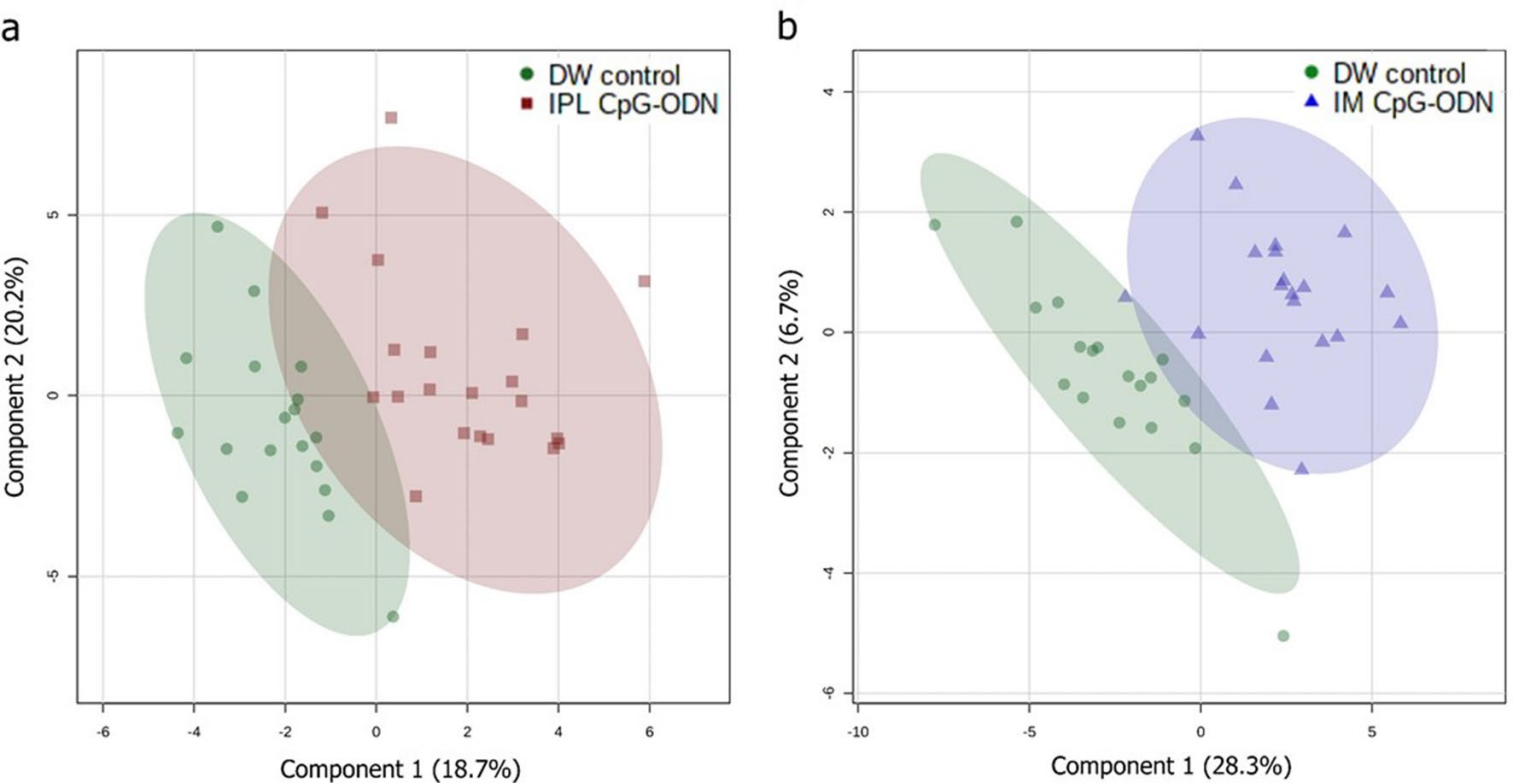
Partial Least Squares-Discriminant Analysis (PLS-DA) of serum metabolites in neonatal chicks 24 h post-treatment. PLS/DA scores plot classifying samples from IPL CpG-ODN treatment vs. DW controls (**a**), and IM CpG-ODN vs. DW controls (**b**), based on measurement of 40 metabolites. DW control, n = 18 (green); IM CpG, n = 20 (blue); IPL CpG, n = 20 (red).

**Figure 4:**
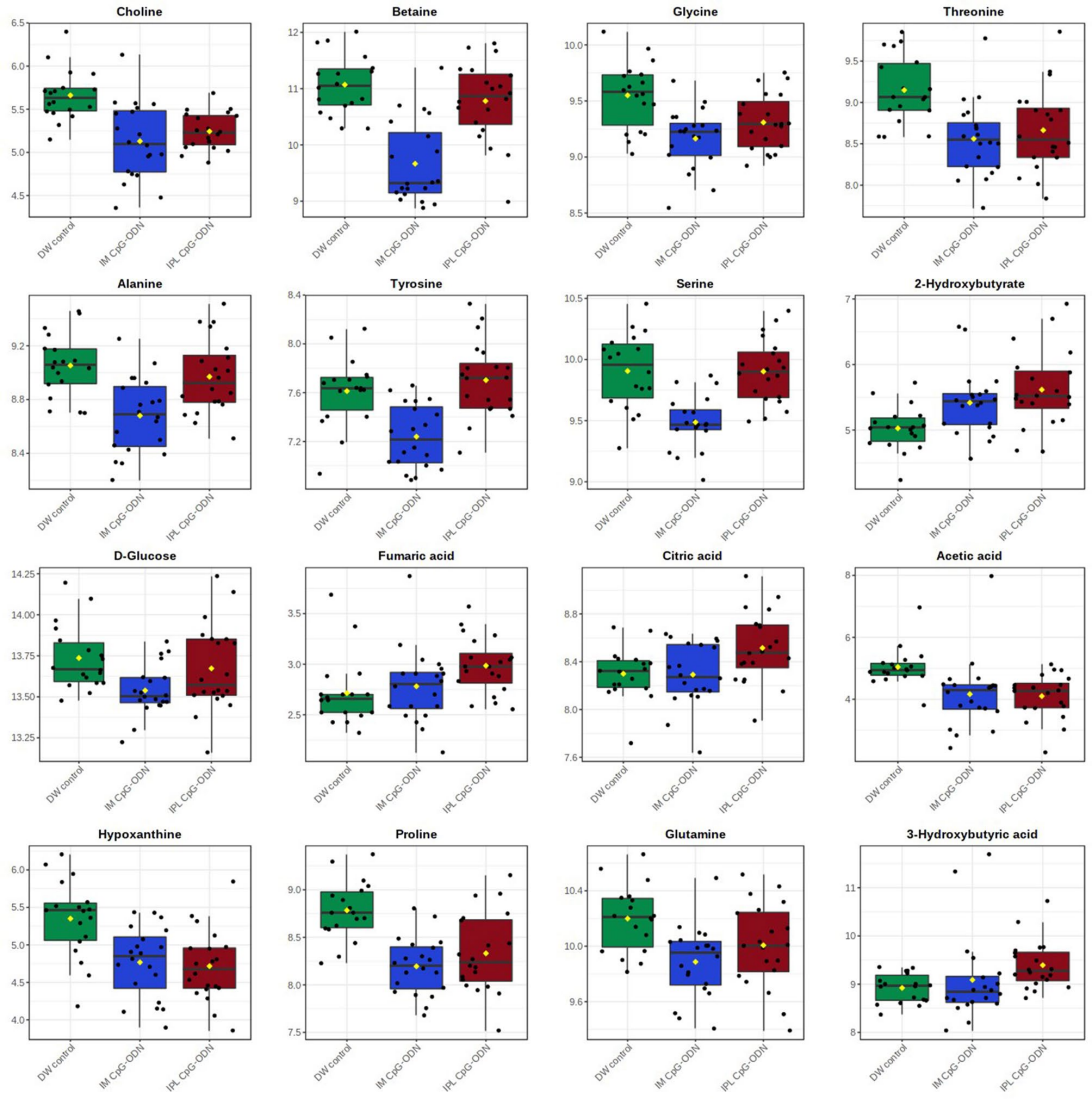
Combined box-and-whisker and dot plots showing the concentrations of selected metabolites in serum from neonatal chicks 24 h post-treatment. DW control, n = 18 (green); IM CpG-ODN, n = 20 (blue) and IPL CpG-ODN, n = 20 (red). Values are log-transformed concentrations in μM. One-way ANOVA results are detailed in supplementary Table S1.

A total of 40 metabolites per experimental group were identified and quantified by NMR. These metabolites were further utilised to find differential biomarkers, by means of univariate and multivariate analysis. First, for quality control measures, unsupervised principle component analysis (PCA) was conducted on all samples together, including pooled samples (Fig. S1). Along with other supporting evidence, this work led to the removal of two outliers from the control group, but not from any experimental group. To further evaluate the group clustering owing to metabolic differences, discriminant analysis was applied per CpG-ODN delivery method vs. DW control. Specifically, a partial least squares discriminant analysis (PLS/DA) model demonstrated a clear separation between IPL CpG-ODN treated chicks and DW control chicks (Fig. 3a). The predictive accuracy using two latent variables was 0.88, the model fitness capability R2Y (cum) was 0.75, and the predictive capability Q2 (cum) was 0.57 after 100 cross-validations. The model significance was further validated via a permutation test with 1000 iterations resulting in *P* < 0.001. Similarly, a separate PLS/DA model also showed some degree of separation between IM CpG-ODN treated chicks and DW control chicks (Fig. 3b). Although the model performance was fine (predictive accuracy = 0.89, R2Y = 0.80, Q2 = 0.55), the second latent variable contributed little to the model (< 7%), and the model significance was marginal at *P* = 0.053 according to the permutation test. The metabolites in the first latent variable of each PLS/DA model corresponded with the most significant metabolites identified in the Student’s *t* tests results. These are detailed in Tables 1 and 2 along with the fold change between treatment and control. They are also described by combined box and whisker and dot plots in Fig. 4.

**Table 1:** Selected Student’s *t*-test results comparing IPL CpG-ODN to DW control. DW controls n = 18; IPL CpG n = 20. Correction for multiple comparisons was conducted using FDR (n = 40 metabolites). Between-group direction and fold-change of the mean raw metabolite concentrations are also presented. IPL CpG-ODN vs. DW control.

Metabolite	IPL/DW	Fold change	*t* test *P* value	FDR—adjusted *P* value
Choline	Down	1.35	1.27E−05	5.08E−04
Acetate	Down	1.93	1.24E−04	0.002
Hypoxanthine	Down	1.56	3.74E−04	0.005
Proline	Down	1.34	6.22E−04	0.006
2-Hydroxybutyrate	Up	1.60	7.85E−04	0.006
3-Hydroxybutyrate	Up	1.45	0.001	0.007
Cytidine	Down	1.56	0.001	0.007
Aspartate	Down	1.30	0.002	0.008
Threonine	Down	1.37	0.003	0.012
Formate	Up	1.49	0.008	0.030
Fumarate	Up	1.19	0.010	0.035
Glycine	Down	1.19	0.011	0.035
Citrate	Up	1.17	0.015	0.047
Uridine	Down	1.32	0.018	0.050
Myo-inositol	Down	1.39	0.020	0.052
Glutamine	Down	1.13	0.037	0.092
Isoleucine	Up	1.15	0.041	0.095
Tryptophan	Down	1.14	0.043	0.095

**Table 2:** Selected Student’s *t*-test results comparing IM CpG-ODN to DW control. DW controls n = 18; IM CpG n = 20 (apart from carnitine, where two missing values were removed). Correction for multiple comparisons was conducted using FDR (n = 40 metabolites). Between-group direction and fold-change of the mean raw metabolite concentrations are also presented. IM CpG-ODN vs. DW control.

Metabolite	IM/DW	Fold change	*t* test *P* value	FDR—adjusted *P* value
Betaine	Down	2.46	7.16E−08	2.86E−06
Proline	Down	1.51	8.45E−07	1.69E−05
Serine	Down	1.35	2.33E−05	3.11E−04
Alanine	Down	1.29	8.64E−05	8.64E−04
Choline	Down	1.41	1.26E−04	9.28E−04
Glycine	Down	1.31	1.86E−04	9.28E−04
Threonine	Down	1.49	1.73E−04	9.28E−04
Tyrosine	Down	1.30	1.48E−04	9.28E−04
Cytidine	Down	1.64	2.31E−04	0.001
Glutamine	Down	1.24	4.54E−04	0.002
Hypoxanthine	Down	1.51	9.11E−04	0.003
Lysine	Down	1.37	0.001	0.004
D-Glucose	Down	1.15	0.001	0.004
Acetate	Down	1.26	0.007	0.019
Aspartate	Down	1.22	0.008	0.020
2-Hydroxybutyrate	Up	1.36	0.009	0.021
Uridine	Down	1.40	0.009	0.021
Valine	Down	1.16	0.017	0.037
Carnitine	Down	1.37	0.029	0.057
Methionine	Down	1.17	0.032	0.068
Tryptophan	Down	1.12	0.037	0.073
Leucine	Down	1.19	0.050	0.093
Malonate	Down	1.25	0.051	0.093
Creatine	Up	1.50	0.068	0.109
Myo-inositol	Down	1.29	0.066	0.109

The metabolic changes resulting from the CpG-ODN treatments are further put into biochemical context and linked to metabolic events as part of the discussion section. Each CpG-ODN treatment was accompanied by significant changes, compared to DW controls, in about fifteen metabolites (*P* < 0.05 after adjustment for multiple comparisons). There was some overlap between the treatments effect, yet unique changes were also recorded for IPL and for IM vs. DW controls. Treatment with CpG-ODN via IM delivery resulted in more significant metabolic changes compared to DW controls (eight metabolites with *P* < 0.001). The most significantly varying metabolites were typically down-regulated, including of amino acids and derivatives, betaine (decrease of 2.5 fold), choline (decrease of 1.4 fold), glucose (decrease of just 1.15 fold) and nucleotide synthesis intermediates (decrease of 1.4–1.65 fold). The only significant elevated metabolite in the IM CpG-ODN group was 2-hydroxybutyrate (1.35 fold, *P* = 0.02), which was increased to a lesser degree and significance than in the IPL CpG-ODN group (1.6 fold, *P* = 0.006 after FDR correction). Delivery of CpG-ODN via IPL also resulted in a unique 1.45-fold increase of the ketone body 3-hydroxybutyrate (*P* = 0.007) and a statistically significant yet quantitatively moderate increase of TCA cycle intermediates, citrate and fumarate (fold change close to 1.2; *P* < 0.05). Delivery of CpG-ODN via IPL also showed a more pronounced decrease of acetate than in the IM treatment (1.9 fold change; *P* = 0.002), while the decrease of nucleotide synthesis intermediates and of some amino acids was similar to that of the IM treatment. An additional comparison between the treatments was conducted via one-way ANOVA. This highlighted differences between IM and IPL delivery methods, mainly in amino acids levels (Table S1).

Pathway enrichment analysis suggested metabolic pathways that were significantly perturbed as a result of the alterations in metabolites between each CpG-ODN group and DW controls (see supplementary Table S2). Despite the rather low coverage per pathway reflected in lower numbers of metabolite hits and lower topological impact, some significant alterations were found in specific pathways involved in energy production and expenditure. Interestingly, the delivery method of the CpG-ODN affected the significance of the pathways that were enriched. IM treatment vs. DW controls consistently exhibited higher statistical significance for pathways involving amino acid metabolism, compared to IPL treatment. On the other hand, IPL was the only delivery method to alter the TCA cycle when compared to DW controls. IPL treatment also showed higher significance for pyruvate metabolism and for glycolysis or gluconeogenesis.

## Discussion

During an immune response, profound metabolic changes occur to facilitate cell signaling, to enhance cytokine protein production, to support rapid B-cell and T-cell division and to fulfill increased energy and anabolic demands^45^. To the best of our knowledge, the present study is the first report on serum metabolic profiling of CpG-ODN induced antimicrobial immunity in chickens. We have extensively reported that standalone CpG-ODN administration via intramuscular (IM)^26–28,30^ or intrapulmonary (IPL)^29,34^ routes can induce antimicrobial immunity against several bacterial pathogens in chickens. Recent metabolomics studies have provided valuable information about the metabolome in various tissues in normal healthy chickens^46,47^. However, no metabolomic data has been collected relating to CpG-ODN induced immune responses in chickens. Therefore, the present study was designed to investigate a potential regulation of metabolic pathways by CpG-ODNs to induce antimicrobial immunity. Here, we used NMR-based metabolomics to explore the potential of immuno-metabolic interactions in the orchestration of CpG-ODN-induced antimicrobial immunity. In doing so, we attempted to identify critical molecules or pathways associated with immunoprotective phenotypes based on their characteristic serum metabolite profiles. Our findings clearly suggest that immune-metabolic interactions are involved in CpG-ODN-mediated immunity. In particular, this study revealed a wide array of differential metabolic signatures in CpG-ODN treated chickens.

**Figure 5:**
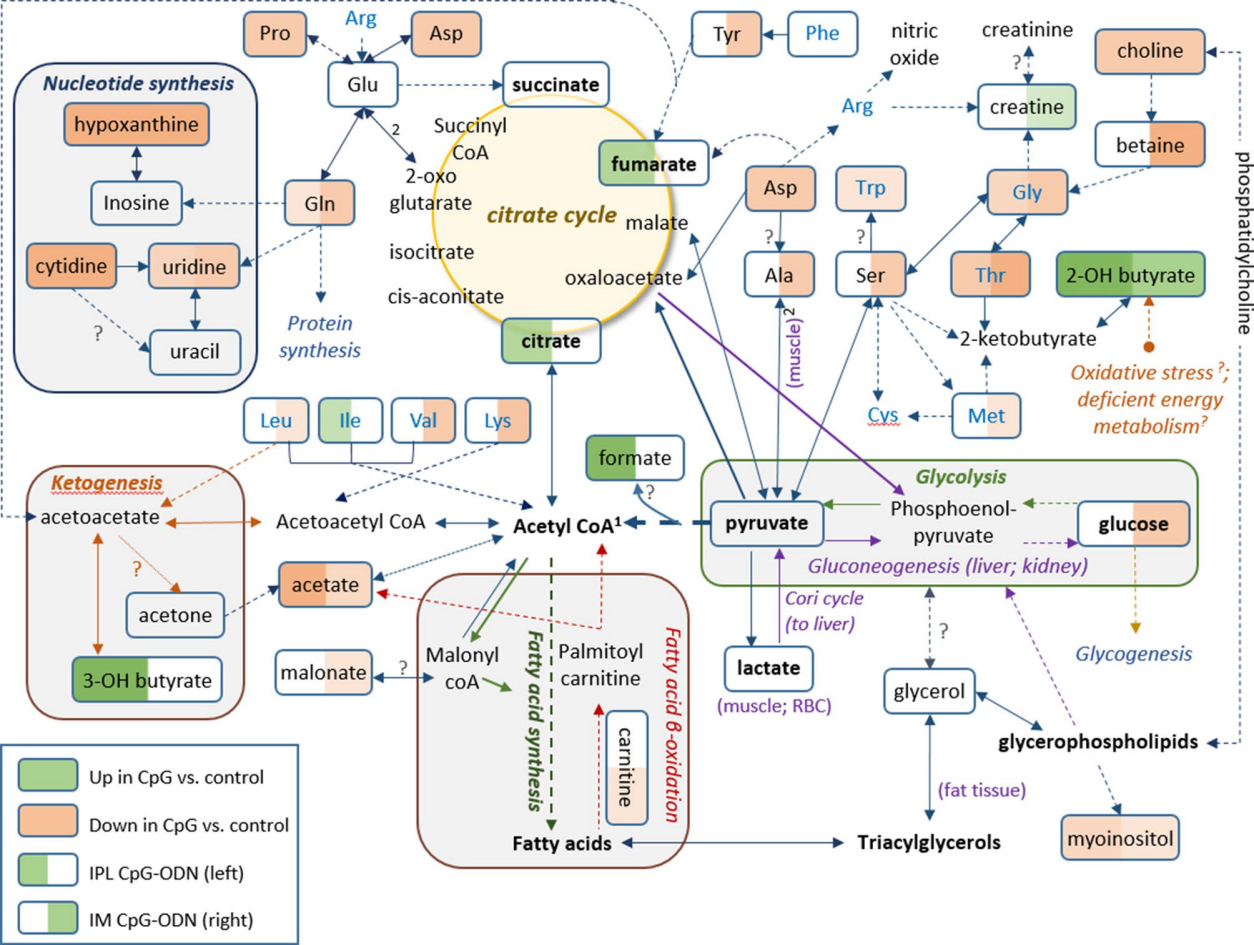
Metabolic pathway map. Pathway map incorporating metabolic differences between IPL CpG-ODN treatment and DW control (left half of each metabolite box) and between IM CpG-ODN treatment and DW control (right half of each metabolite box). According to the Student’s *t *test (Table 1, 2), metabolite boxes with a green background were significantly higher in CpG-ODN vs. DW controls, while metabolite boxes with a red background were lower in CpG-ODN compared to DW controls, 24 h post treatment. Higher levels of significance and fold change are reflected by a darker color, and lower levels and trends by a lighter color. Uncolored part of a box indicates metabolites that did not show any trend of change in concentrations between CpG-ODN and DW controls. Full arrows represent one-step metabolic conversion, and broken arrows depict at least two steps between a precursor and its derivative. Essential amino acids are in blue text, and metabolic events or location are in italic text with different colors^1^. In animals, acetyl-CoA cannot be used as a substrate for gluconeogenesis^2^. The two reactions depend on each other. ^?^ Unestablished in chickens.

In the present study, chickens treated with intramuscular (IM) CpG-ODNs were better protected against bacterial infection than the intrapulmonary (IPL) group (Figs. 1, 2). This difference between the two CpG-ODN delivery methods corresponded well with the observed alteration in metabolic profile between the treatment groups, despite of natural variation in response between birds in the same group (Fig. 4). To better illustrate the metabolic alterations within their biochemical context, Fig. 5 presents a metabolic pathways map incorporating the t test results comparing each treatment to control (Tables 1, 2). It is well known that CpG-ODN causes the immune system's stimulation, resulting in pro-inflammatory cytokine secretion^48^ and activation of immune cells such as monocytes, macrophages, and lymphocytes. We recently reported an increased number of mononuclear cells infiltrating into the lungs of chickens within 24 h of CpG-ODN treatment^2,29,33^. Given that immune cells must grow and divide rapidly upon activation, despite lacking nutrient stores, they require energy-rich resources, including sugars, amino acids, and fatty acids present in the extracellular environment^49^. Therefore it is expected that the levels of these metabolites, during immune activation, would potentially decrease in the serum^50^. In particular, immune activation requires amino acids for protein synthesis, nucleotides for DNA/RNA synthesis, sugars for energy production and fatty acids for lipid membrane production. As immune cell activation is certainly metabolically demanding, we assume that the metabolomics changes in serum that we observed in the present study could be the result of increased enrichment and activation of immunological niches^2,29^, leading to enhanced uptake of metabolites by cells during immunological responses. Since blood bathes every organ and every tissue in the body, it essentially reflects the net metabolic changes resulting from the physiological and metabolic needs or stresses in different tissues in the animal body^72^. However, our serum metabolomics data do not distinguish between the possibilities of differential use or synthesis or uptake of metabolites in various tissues following the CpG-ODN administration. Additional studies and techniques will be required to investigate the complex issues of differentiating between the use, synthesis, and uptake of substances.

Several studies have reported that amino acids are critical in immune cell proliferation and function^51^. A study in mice demonstrated that serine and glycine play an important role in T cell proliferation and function^50^. In our study, we found a significant decrease in serum levels of essential and also non-essential amino acids. Altered levels of essential amino acids which are required for protein synthesis, included threonine (35% decrease in IPL treatment and 50% decrease in IM treatment), glycine (20–30% decrease in both treatments), and lysine (35% decrease, only in IM treatment). A recent study demonstrated that the metabolism of a single amino acid (proline) can have a profound effect on the immune responses to pathogens^52^. This study reported that mitochondrial proline catabolism controls innate immunity in Caenorhabditis elegans by regulating reactive oxygen species (ROS) homeostasis. In both nematodes and birds, antimicrobial ROS produced by macrophages^53^ and neutrophils^54^ aids in bacterial killing. In our study, we found a substantial reduction in the serum proline levels that corresponded with increased antimicrobial immunity in both the IPL CpG-ODN and IM CpG-ODN groups. We hypothesize that the CpG-ODN induced increased leukocytes in various immunological niches^2,33,34^, and enhanced activation of macrophages and heterophils [equivalent to mammalian neutrophils], which probably led to greater utilization of serum proline. Further studies to test the role of proline utilization and CpG-ODN induced antimicrobial immunity would be of great interest.

Apart from their role as building blocks in protein synthesis and providing alternative substrates to the TCA cycle, amino acids are also precursors in nucleotide synthesis and play a vital role in cell proliferation. Specifically, glutamine is a key amino acid that is further metabolised into purines and downstream nucleotides to generate DNA and RNA. Increased uptake of glutamine and its precursors aspartate and also proline was observed after administration of CpG-ODN via IM and also IPL. Within the purine metabolism pathway, the three intermediates hypoxanthine, cytidine and uridine showed a uniform decrease across the CpG treatments.

In addition to the amino acid changes noted above, we also observed reduced acetate levels in serum upon the administration of CpG-ODN. The source of acetic acid might be either a catabolism product of ketone bodies, or metabolism of acetyl-CoA, an essential metabolite of carbohydrate and fatty acid metabolism^55^. As mentioned earlier, cell proliferation also increases the demands for fatty acid synthesis, hence acetic acid might be utilised in this direction. On the other hand, fatty acid oxidation provides energy to the cellular activities when in need through β-oxidation in mitochondria or peroxisomes. An increased fatty acid uptake in the liver results in ketogenesis, where fatty acids undergo incomplete oxidation^56^. Lower serum acetate levels resulting from CpG-ODN administration might be indicating that immune cells must start dividing and that there is a need for extra energy. As a result, fatty acids started to undergo oxidation to provide that extra energy. Acetate also appears to play a signaling role in the immune system. A recent report has shown how acetate promotes T-cell effector functions^57^ through epigenetic modifications and that rapidly dividing cells (including both tumor and immune cells) can use acetate (instead of glucose) as an alternative fuel.

We also found that CpG-ODN administration by both IPL and IM resulted in increased levels of 2-OH butyrate, which can originate in several amino acids via 2-ketobutyrate. In humans 2-OH butyrate is related to lipid oxidation, oxidative stress and deficient energy metabolism^58^. Interestingly, serum levels of 2-OH butyrate increased by 60% after the IPL treatment and only by 35% in the IM treatment. Further metabolic differences were observed between the two administration routes, with a slight decrease in glucose measured only in the IM group, while a 45% increase in the ketone body 3-OH butyrate was observed only in the IPL-CpG-ODN group, indicating ketogenesis was being used to provide for higher energy demands. In addition, an increase in TCA cycle intermediates was recorded only after IPL administration, although the lactate-to-pyruvate ratio did not differ between the experimental groups. Altogether, these data suggest differences in the extent and rate of energy metabolism adaptations, potentially due to systemic vs. mucosal targeting by IM and IPL CpG-ODN administration methods, respectively.

Beyond its function as an energy source, a previous study in cattle reported that beta-hydroxybutyrate (3-OH butyrate) abrogates the antimicrobial function of neutrophils against E. coli^59^. Several other studies showed that 3-hydroxybutyrate upregulates mRNA abundance of proinflammatory cytokines such as IL-1β in mouse macrophages^60^, IL-8 in bovine liver^61^, and several pro-inflammatory cytokines in calf hepatocytes^62^. The cytokine mRNA data should be interpreted cautiously, as some cytokines such as IL-1β are highly regulated post-translationally^63^. In our previous studies, we found a significant increase in the expression of several proinflammatory cytokines, including IL-1β and IL-6 following CpG-ODN administration^2,33^. In the present study, we found an increased serum level of 3-hydroxybutyrate in the IPL CpG-ODN group, but how this would affect cytokines at the protein level needs further studies.

Our metabolomic analysis also highlighted two crucial metabolites, choline and betaine. Serum choline levels were reduced by 35–40% in the CpG-ODN treated birds while betaine levels were significantly lower (2.5-fold) only in the birds that received intramuscular CpG-ODN treatment. Choline is a precursor in the synthesis of acetylcholine, phosphorylcholine, and is an important intermediate in phospholipid metabolism. It is well documented that there is increased consumption of phosphorylcholine under severe oxidative and systemic inflammatory conditions^64^. Choline is oxidized to betaine, and both are linked to the folate-dependent one-carbon metabolism^65^. In chickens, choline plays a significant role in improving the humoral and cellular immunity^66^, and betaine has been shown to increase lymphocyte infiltration in infected mucosa. Betaine also enhances phagocytic and nitric oxide production by blood monocytes and heterophils^67^. A recent study in cattle reported that T cell proliferation was linearly enhanced in vitro with increasing doses of choline^68^. Reduced choline levels in our study could also be contributing to the low betaine levels, however betaine is also supplemented via the feed due to its nutritional importance.

As noted earlier, increased utilization of choline, betaine, and proline by immune cells can enhance the antimicrobial activity of various immune cells. Additionally, given that choline, betaine, and proline are effective osmolytes^69^, their reduced levels in the serum would prevent bacterial proliferation due to a reduced accumulation of osmoprotectants (betaine, choline, and proline), which play an essential role in bacterial growth such as in the case of E. coli^69^ and Staphylococcus aureus^70^. It has been reported that betaine provides the best osmotic protection to E. coli^69^ growth followed by choline (which is converted to betaine) and proline^69^. Interestingly, in our study, we found that IM CpG-ODN administration dramatically reduced the serum levels of betaine followed by proline and choline, and provided better protection against E. coli compared to IPL-CpG-ODN, which only showed significantly reduced serum levels of choline and proline but not betaine. More significant reduction of bacterial growth supporting osmoprotectants in IM CpG-ODN vs. IPL CpG-ODN correlates well with the rank order of their protection data (IM-vs. IPL-CpG-ODN).

Overall, the metabolomic data from this study suggest that CpG-ODN-mediated antimicrobial immunity involves a number of significant metabolic changes in the host that enhance immunity and antagonize microbial proliferation in the host. CpG-ODN induced metabolomics data generated by the current study provides a unique and little-appreciated approach to identify regulatory molecules or pathways that give protective immunity to chickens against bacterial infections. The metabolites highlighted in the present study can help future targeted studies better understand antimicrobial metabolomic profiles and pinpoint the key molecules or pathways contributing to immunity.

## Materials and methods

### Housing and maintenance of experimental chickens

This work was carried out in compliance with the ARRIVE guidelines. The animal study was approved by the Animal Research Ethics Board, University of Saskatchewan (protocol number 20070008) and adhered to the guidelines of the Canadian Council on Animal Care. Euthanasia was performed by cervical dislocation following the AVMA guidelines for the euthanasia of animals. Day-old broiler chickens (Ross 308 strain) were obtained from a commercial hatchery in Saskatchewan. Groups of chicks were allocated randomly into an animal isolation room at the Animal Care Unit, Western College of Veterinary Medicine in Saskatoon, Saskatchewan, and chicks were maintained following the procedure as described earlier^2,33^. Briefly, water and commercial broiler starter ration (23% crude protein, 1% calcium, 0.45% available phosphorous) were provided ad libitum. Air from each room was exhausted through a HEPA filter, and non-recirculated intake air was provided at a rate of 15–20 air changes/hr. Air pressure differentials and strict sanitation were maintained in this isolation facility. Broilers were raised at 32 °C for the first 7 days of life (average weight ~ 180 g); after that, the temperature was decreased by 0.5 °C per day until a room temperature of 27.5 °C was reached. Light (30 lx) was provided for 24 h/d during days 0 to 2 (post-hatch). Darkness was introduced at 3 d post-hatch with 1 h of dark added daily until 4 h of darkness was achieved.

### CpG-ODN delivery

The CpG-ODN [TCGTCGTTGTCGTTTTGTCGTT (2007)] was free of endotoxin and produced with a phosphorothioate backbone (Operon Biotechnologies, Inc; Huntsville, AL, USA). Synthetic CpG-ODN was diluted in sterile DW and delivered by the IPL route. Briefly, the CpG-ODN solution was aerosolized as micro-droplets (particle size of 0.5–5 µm) using a Compressor Nebulizer (705–470) unit (AMG Medical Inc; Montreal, QC, Canada) in a closed 0.036 m^3^ acrylic chamber containing 60 birds for 30 min (6 mg CpG-ODN/ chamber) that maintained atmospheric oxygen exchange. The control group of birds (n = 60) were aerosolized with DW for 30 min in the acrylic chamber using a similar compressor nebulizer. Another group of birds (n = 60) were administered with CpG-ODN (50 μg/100 μl/bird) by IM injection to the left thigh. The temperature was maintained at 28–30 °C in the acrylic chamber during the administration of CpG-ODN or DW.

### E. coli culture and animal model

In order to confirm the immune protection induced by CpG-ODN delivery, a parallel *E. coli* challenge study was performed to the birds. A field isolate of *E. coli* from a turkey with septicemia was used as the challenge strain according to our previously established animal model^29,30^. The *E. coli* belonged to serogroup O2 was nonhemolytic, serum resistant, aerobactin producing and had K1 capsule with type I pili^28^. Aliquots of the bacterial isolate were stored at − 80 °C in brain heart infusion broth (Difco, Detroit, Mich.) supplemented with 25% (wt/vol) glycerol (VWR Scientific Inc., Montreal, QC, Canada). In order to challenge the birds, bacteria were cultured on 5% Columbia sheep blood agar for 18–24 h at 37 °C. One colony of *E. coli* was added to 100 mL of Luria broth (Difco LB broth, Miller, Becton Dickinson and Company; Sparks, MD, USA) in a 250 mL Erlenmeyer flask. The culture was grown at 37 °C for 16–18 h, shaking at 150 rpm. This stationary phase culture contained approximately 1 × 10^9^ colony forming units (CFU) of bacteria per mL, which was then further diluted into saline to the concentration of bacteria required to challenge birds. The *E. coli* challenge dose was confirmed by plating serial dilutions of the diluted culture in duplicate on 5% Columbia sheep blood agar plates, incubating for 18 h at 37 °C, then counting the number of colonies.

The *E. coli* challenge study was performed according to the well-established animal model that we documented earlier^26,29^. Briefly, on the second-day post-treatment, the birds in each group (n = 40) were challenged with 1 × 10^5^ CFU (n = 20) or 1 × 10^6^ CFU (n = 20) of bacteria per bird, subcutaneously in the neck. They were closely monitored three times a day for the most critical period of three days post-challenge and two times a day thereafter until seven days post-challenge. Each bird was observed for clinical signs, and a daily clinical score was assigned: 0 = normal; 0.5 = slightly abnormal appearance, slow to move; 1 = depressed, reluctant to move; 1.5 = reluctant to move, may take a drink and peck some; 2 = unable to stand or reach for food or water; and 3 = found dead. Birds that received a clinical score of 2 were euthanized by cervical dislocation. At the end of the trial, each bird was given a cumulative clinical score (CCS) as a sum of daily clinical scores, as previously described^26,29^.

When chicks were found dead or euthanized, they were necropsied immediately. All remaining birds were euthanized on day seven post-challenge. Air sac swabs were obtained from dead or euthanized birds and a semi-quantitative estimate of *E. coli* isolation was conducted on 5% Columbia sheep blood agar by the quadrant streaking method in which bacterial colonies thinning occurs as streaking goes clockwise from quadrant 1 to quadrant 4. Bacterial growth on these cultures were recorded on a scale from 0 to 4 + , where 0 = no growth; few = less than 5 colonies; 1 +  = growth of bacteria on quadrant 1; 2 +  = growth of bacteria on quadrants 1 and 2; 3 +  = growth of bacteria on quadrants 1, 2 and 3; and 4 +  = growth of bacteria on all quadrants 1–4 as reported previously^71^*.* For the statistical analysis on bacterial loads, birds were divided into two categories in each group. In category one, birds with low bacterial loads [zero or few (~ 5) bacterial colonies in the blood agar] were included. Whereas, category two included birds with high bacterial loads [bacterial growth in various quadrants; 1 + , 2 + , 3 + & 4 + in the blood agar].

### Metabolomics analysis of serum

#### Sample collection

Twenty chicks from each group were euthanized 24 h post-CpG-ODN treatment in the morning between 9 am to 10 a.m. Blood was immediately collected into serum tubes by severing the necks of the chicks with a sharp pair of scissors. After about 30 min of blood collection, the clotted blood samples were then centrifuged at 1000*g* force for 15 min, and serum was separated into 1.5 mL microcentrifuge tubes. Serum samples were stored at − 80 °C, transported on dry ice to The Metabolomics Innovation Centre (TMIC) facility at the University of Alberta in Edmonton, and stored at − 80 °C until further analysis.

#### Sample preparation and NMR spectroscopy

Serum samples were thawed on ice and prepared in two batches according to a randomization template, with the addition of pooled samples for quality control. Plasma and serum samples contain a significant concentration of large molecular weight proteins and lipoproteins, which can seriously compromise the quality of ^1^H-NMR spectra though the generation of intense, broad lines that interfere with the identification and quantification of lower abundance metabolites. Deproteinization can eliminate these peaks. Deproteinization of the serum samples was done by centrifugation and ultrafiltration using 3-kDa cut-off centrifuge filter units (Microcon YM-3; Sigma-Aldrich, St. Louis, MO), following a previously reported deproteinization procedure^72^. The deproteinized serum samples (280 μL) were then transferred to a 1.5 mL micro centrifuge tube followed by the addition of 70 μL standard NMR buffer solution (1 mM DSS (disodium-2, 2-dimethyl-2-silapentane-5-sulphonate), in 10% D_2_O). These samples (a total volume of 350 μL) were then transferred to a 3 mm NMR tube for spectral analysis. All ^1^H-NMR spectra were collected on a Bruker Avance III Ascend 700 MHz spectrometer with a 5 mm cryo-probe (Bruker Biospin, Rheinstetten, Germany). ^1^H-NMR spectra were acquired at 25 °C using the first transient of the noesy-presaturation pulse sequence, which was chosen for its high degree of quantitative accuracy^73^. Spectra were collected with 128 transients using a 4 s acquisition time and a 1 s recycle delay.

#### NMR compound identification and quantification

Before spectral analysis, all free induction decays (FIDs) were zero-filled to 240 k data points, and a line broadening of 0.5 Hz was applied. The methyl singlet of the added DSS served as an internal standard for chemical shift referencing (set to 0.00 ppm) and for quantification. All ^1^H-NMR spectra were processed and imported into the Chenomx NMR Suite 8.1 software (Edmonton, Canada). The Chenomx NMR Suite software allows for a quantitative analysis of an NMR spectrum by manually fitting spectral signatures from an internal database to the spectrum. Specifically, the spectral fitting for metabolite was done using the standard Chenomx 700 MHz metabolite library. Most of the visible peaks are annotated with a compound name. Each spectrum was processed and analyzed by at least two experienced NMR spectroscopists to minimize compound misidentification and misquantification. Forty metabolites passed the NMR quality measures and underwent further statistical analysis.

#### Data processing and statistical analysis

The MetaboAnalyst 4.0 package^74^ was used for statistical analysis of the metabolomics data. Data were log-transformed prior to univariate analysis, and also autoscaled prior to multivariate analysis. Principal Component Analysis (PCA) was performed for quality-control assessment. Partial Least Squares-Discriminant Analysis (PLS/DA) was used to classify samples and suggest potential biomarkers for treatment effect. Several univariate analysis tests were also employed. In particular, analysis of variance (ANOVA) was conducted to compare metabolite levels between all three groups, with Tukey’s HSD post-hoc analysis to indicate significant pairs. Student’s *t* test was used to compare between two experimental groups in terms of fold-change analysis and assessment of significance with regard to metabolite differences. In all tests, *P* values were further corrected for multiple comparisons by applying the Benjamini–Hochberg method of false discovery rate (FDR), and considering an FDR-adjusted *P* value of 0.1 as a threshold for inclusion in tables and figures. Pathway enrichment analysis was performed in the MetaboAnalyst 4.0 software on log-transformed and auto scaled data, against the Gallus Gallus pathway database. For each two-group comparison, it consisted of an ANCOVA test with the use of a relative-betweenness centrality algorithm for pathway topology analysis.

The significance of the observed differences in chick survival and the cumulative clinical score (CCS) were analyzed using GraphPad Prism 8.0 (GraphPad Software Inc., San Diego, CA) with a significance level of *P* < 0.05. The survival data and bacterial scores of both 1 × 10^5^ CFU and 1 × 10^6^ CFU of *E. coli* challenge were combined for clarity of analysis and presentation. The level of significance with regard to differences among groups in survival patterns and median survival times were analyzed using the log-rank test and chi-square statistic. Clinical scores assigned at each time point were summed up to 7 days post-challenge to generate CCS and thereby daily mean CCSs were calculated for each group. Two-way ANOVA was performed with Dunnett’s multiple comparison tests to compare the significant differences in mean CCS. For the statistical analysis on bacterial loads (quadrant streaking method), birds in each group were divided into two categories (low or high bacterial load). A chi-square test of independence was performed to examine the relationship between the CpG treatment method and the ability to recover viable bacteria based on this categorization. The results were interpreted with a statistical significance of *P* < 0.05.

## Supplementary Information

Supplementary Information

## References

[1] Coffman RL, Sher A, Seder RA (2010). Vaccine adjuvants: Putting innate immunity to work. Immunity.

[2] Goonewardene, K., Ahmed, K.A., Gunawardana, T. *et al.* Mucosal delivery of CpG-ODN mimicking bacterial DNA via the intrapulmonary route induces systemic antimicrobial immune responses in neonatal chicks. *Sci. Rep.* **10**, 5343. 10.1038/s41598-020-61683-y (2020). 10.1038/s41598-020-61683-y PMC7093454 32210244

[3] Gursel, M. & Gursel, I. Development of CpG ODN based vaccine adjuvant formulations. In *Vaccine Design* 289–298 (Humana Press, New York, NY, 2016). 10.1007/978-1-4939-3389-1_20. 10.1007/978-1-4939-3389-1_20 27076306

[4] Gursel M, Klinman DM, Mestecky J (2015). Chapter Use of CpG oligonucleotides as mucosal adjuvants. Mucosal Immunology.

[5] Krieg AM (2002). CpG motifs in bacterial DNA and their immune effects. Annu. Rev. Immunol..

[6] Staeheli P, Puehler F, Schneider K, Göbel TW, Kaspers B (2001). Cytokines of birds: Conserved functions—A largely different look. J. Interferon Cytokine Res..

[7] Finlay BB, Hancock REW (2004). Can innate immunity be enhanced to treat microbial infections?. Nat. Rev. Microbiol..

[8] Aderem A, Ulevitch RJ (2000). Toll-like receptors in the induction of the innate immune response. Nature.

[9] Brownlie R, Allan B (2011). Avian toll-like receptors. Cell Tissue Res..

[10] Brownlie R (2009). Chicken TLR21 acts as a functional homologue to mammalian TLR9 in the recognition of CpG oligodeoxynucleotides. Mol. Immunol..

[11] Hartmann G (2000). Delineation of a CpG phosphorothioate oligodeoxynucleotide for activating primate immune responses in vitro and in vivo. J. Immunol..

[12] Keestra AM, de Zoete MR, Bouwman LI, van Putten JPM (2010). Chicken TLR21 Is an Innate CpG DNA Receptor Distinct from Mammalian TLR9. J. Immunol..

[13] Krieg AM (2008). Toll-like receptor 9 (TLR9) agonists in the treatment of cancer. Oncogene.

[14] Yeh D-W (2013). Toll-like receptor 9 and 21 have different ligand recognition profiles and cooperatively mediate activity of CpG-oligodeoxynucleotides in zebrafish. PNAS.

[15] Bode C, Zhao G, Steinhagen F, Kinjo T, Klinman DM (2011). CpG DNA as a vaccine adjuvant. Expert Rev. Vaccines.

[16] Hanagata N (2017). CpG oligodeoxynucleotide nanomedicines for the prophylaxis or treatment of cancers, infectious diseases, and allergies. Int. J. Nanomed..

[17] Shirota H, Tross D, Klinman DM (2015). CpG oligonucleotides as cancer vaccine adjuvants. Vaccines.

[18] Zhang H, Gao X-D (2017). Nanodelivery systems for enhancing the immunostimulatory effect of CpG oligodeoxynucleotides. Mater. Sci. Eng. C Mater. Biol. Appl..

[19] Adamsson J (2006). Novel immunostimulatory agent based on CpG oligodeoxynucleotide linked to the nontoxic B subunit of cholera toxin. J. Immunol..

[20] Weiner GJ, Liu H-M, Wooldridge JE, Dahle CE, Krieg AM (1997). Immunostimulatory oligodeoxynucleotides containing the CpG motif are effective as immune adjuvants in tumor antigen immunization. Proc. Natl. Acad. Sci. USA.

[21] Meng W, Yamazaki T, Nishida Y, Hanagata N (2011). Nuclease-resistant immunostimulatory phosphodiester CpG oligodeoxynucleotides as human Toll-like receptor 9 agonists. BMC Biotechnol..

[22] Cho HC (2008). Cancer immunotherapeutic effects of novel CpG ODN in murine tumor model. Int. Immunopharmacol..

[23] Nichani AK (2004). In vivo immunostimulatory effects of CpG oligodeoxynucleotide in cattle and sheep. Vet. Immunol. Immunopathol..

[24] Jørgensen JB, Johansen L-H, Steiro K, Johansen A (2003). CpG DNA induces protective antiviral immune responses in Atlantic salmon (*Salmo salar* L.). J. Virol..

[25] Dalloul RA (2004). In vivo effects of CpG oligodeoxynucleotide on eimeria infection in chickens. Avian Dis..

[26] Gomis S (2004). Protection of neonatal chicks against a lethal challenge of *Escherichia coli* using DNA containing cytosine-phosphodiester-guanine motifs. Avian Dis..

[27] Taghavi A (2008). Protection of neonatal broiler chicks against salmonella typhimurium septicemia by DNA containing CpG motifs. Avian Dis..

[28] Gomis S (2003). Protection of chickens against *Escherichia coli* infections by DNA containing CpG motifs. Infect. Immunol..

[29] Goonewardene KB (2017). Intrapulmonary delivery of CpG-ODN microdroplets provides protection against *Escherichia coli* septicemia in neonatal broiler chickens. Avian Dis..

[30] Gunawardana T (2014). Protection of neonatal broiler chickens following in ovo delivery of oligodeoxynucleotides containing CpG motifs (CpG-ODN) formulated with carbon nanotubes or liposomes. Avian Dis..

[31] He H, Lowry VK, Swaggerty CL, Ferro PJ, Kogut MH (2005). In vitro activation of chicken leukocytes and in vivo protection against Salmonella enteritidis organ invasion and peritoneal *S. enteritidis* infection-induced mortality in neonatal chickens by immunostimulatory CpG oligodeoxynucleotide. FEMS Immunol. Med. Microbiol..

[32] MacKinnon KM (2009). In ovo treatment with CpG oligodeoxynucleotides decreases colonization of Salmonella enteriditis in broiler chickens. Vet. Immunol. Immunopathol..

[33] Gunawardana T (2019). Synthetic CpG-ODN rapidly enriches immune compartments in neonatal chicks to induce protective immunity against bacterial infections. Sci. Rep..

[34] Gunawardana T (2020). CpG-ODN induces a dose-dependent enrichment of immunological niches in the spleen and lungs of neonatal chicks that correlates with the protective immunity against *Escherichia coli*. J. Immunol. Res..

[35] Cheng S-C (2014). mTOR- and HIF-1α-mediated aerobic glycolysis as metabolic basis for trained immunity. Science.

[36] Gubser PM (2013). Rapid effector function of memory CD8+ T cells requires an immediate-early glycolytic switch. Nat. Immunol..

[37] Pearce EL (2009). Enhancing CD8 T cell memory by modulating fatty acid metabolism. Nature.

[38] Jha AK (2015). Network integration of parallel metabolic and transcriptional data reveals metabolic modules that regulate macrophage polarization. Immunity.

[39] Newsholme P, Curi R, Gordon S, Newsholme EA (1986). Metabolism of glucose, glutamine, long-chain fatty acids and ketone bodies by murine macrophages. Biochem. J..

[40] Donnelly RP, Finlay DK (2015). Glucose, glycolysis and lymphocyte responses. Mol. Immunol..

[41] Frauwirth KA, Thompson CB (2004). Regulation of T lymphocyte metabolism. J. Immunol..

[42] van Stipdonk MJB (2003). Dynamic programming of CD8^+^ T lymphocyte responses. Nat. Immunol..

[43] Wang R (2011). The transcription factor Myc controls metabolic reprogramming upon T lymphocyte activation. Immunity.

[44] Guthrie LA, McPhail LC, Henson PM, Johnston RB (1984). Priming of neutrophils for enhanced release of oxygen metabolites by bacterial lipopolysaccharide. Evidence for increased activity of the superoxide-producing enzyme. J. Exp. Med..

[45] Kelly B, O’Neill LA (2015). Metabolic reprogramming in macrophages and dendritic cells in innate immunity. Cell Res..

[46] Jastrebski SF, Lamont SJ, Schmidt CJ (2017). Chicken hepatic response to chronic heat stress using integrated transcriptome and metabolome analysis. PLoS ONE.

[47] Le Roy, C. I., Mappley, L. J., La Ragione, R. M., Woodward, M. J. & Claus, S. P. NMR-based metabolic characterization of chicken tissues and biofluids: A model for avian research. *Metabolomics* **12** (2016). 10.1007/s11306-016-1105-7 PMC5025519 27729831

[48] Patel BA (2008). Oligodeoxynucleotides containing CpG motifs (CpG-ODN) predominantly induce Th1-type immune response in neonatal chicks. Dev. Comp. Immunol..

[49] Ganeshan K, Chawla A (2014). Metabolic regulation of immune responses. Annu. Rev. Immunol..

[50] Ma EH (2017). Serine is an essential metabolite for effector T cell expansion. Cell Metab..

[51] Grohmann U (2017). Amino-acid sensing and degrading pathways in immune regulation. Cytokine Growth Factor Rev..

[52] Tang H, Pang S (2016). Proline Catabolism Modulates Innate Immunity in *Caenorhabditis elegans*. Cell Rep..

[53] Abuaita BH, Schultz TL, O’Riordan MX (2018). Mitochondria-derived vesicles deliver antimicrobial reactive oxygen species to control phagosome-localized *Staphylococcus aureus*. Cell Host Microbe.

[54] Phan QT (2018). Neutrophils use superoxide to control bacterial infection at a distance. PLoS Pathog..

[55] Human Metabolome Database: Search Results for metabolite. http://www.hmdb.ca/unearth/q?utf8=%E2%9C%93&query=acetate&searcher=metabolites&butt=.

[56] Nguyen P (2008). Liver lipid metabolism. J. Anim. Physiol. Anim. Nutr..

[57] Leone RD (2019). Glutamine blockade induces divergent metabolic programs to overcome tumor immune evasion. Science.

[58] Gall WE (2010). α-Hydroxybutyrate is an early biomarker of insulin resistance and glucose intolerance in a nondiabetic population. PLoS ONE.

[59] Grinberg N, Elazar S, Rosenshine I, Shpigel NY (2008). Beta-hydroxybutyrate abrogates formation of bovine neutrophil extracellular traps and bactericidal activity against mammary pathogenic *Escherichia coli*. Infect. Immun..

[60] Swartz, T., Mamedova, L. & Bradford, B. Beta-Hydroxybutyrate Alters the mRNA Cytokine Profile from Mouse Macrophages Challenged with Streptococcus uberis. *Kansas Agric. Exp. Stn. Res. Rep.* **5** (2019).

[61] Zarrin M, Wellnitz O, van Dorland HA, Bruckmaier RM (2014). Induced hyperketonemia affects the mammary immune response during lipopolysaccharide challenge in dairy cows. J. Dairy Sci..

[62] Shi X (2014). β-Hydroxybutyrate activates the NF-κB signaling pathway to promote the expression of pro-inflammatory factors in calf hepatocytes. Cell. Physiol. Biochem..

[63] Youm Y-H (2015). The ketone metabolite β-hydroxybutyrate blocks NLRP3 inflammasome-mediated inflammatory disease. Nat. Med..

[64] Guleria A (2016). NMR based serum metabolomics reveals a distinctive signature in patients with Lupus Nephritis. Sci. Rep..

[65] Ueland PM (2011). Choline and betaine in health and disease. J. Inherit. Metab. Dis..

[66] Swain BK, Johri TS (2000). Effect of supplemental methionine, choline and their combinations on the performance and immune response of broilers. Br. Poult. Sci..

[67] Klasing KC, Adler KL, Remus JC, Calvert CC (2002). Dietary betaine increases intraepithelial lymphocytes in the duodenum of coccidia-infected chicks and increases functional properties of phagocytes. J. Nutr..

[68] Garcia M, Mamedova LK, Barton B, Bradford BJ (2018). Choline regulates the function of bovine immune cells and alters the mRNA abundance of enzymes and receptors involved in its metabolism in vitro. Front. Immunol..

[69] Cayley S, Lewis BA, Record MT (1992). Origins of the osmoprotective properties of betaine and proline in Escherichia coli K-12. J. Bacteriol..

[70] Graham JE, Wilkinson BJ (1992). Staphylococcus aureus osmoregulation: Roles for choline, glycine betaine, proline, and taurine. J. Bacteriol..

[71] Hoeprich, P. D. *Infectious diseases: A guide to the understanding and management of infectious processes*. (Medical Dept., Harper & Row, 1972).

[72] Psychogios N (2011). The human serum metabolome. PLOS ONE.

[73] Saude EJ, Slupsky CM, Sykes BD (2006). Optimization of NMR analysis of biological fluids for quantitative accuracy. Metabolomics.

[74] Chong J (2018). MetaboAnalyst 4.0: Towards more transparent and integrative metabolomics analysis. Nucleic Acids Res..

