# Supplementary material for: CpG-ODN induced antimicrobial immunity in neonatal chicks involves a substantial shift in serum metabolic profiles
Source: Sci Rep. 2021 Apr 27;11:9028. doi: 10.1038/s41598-021-88386-2 (PMC8079682; doi:10.1038/s41598-021-88386-2)
Supplement: Supplementary file 1 — Supplementary Information [file 41598_2021_88386_MOESM1_ESM.pdf]

Supplementary information

CpG-ODN induced antimicrobial immunity in neonatal chicks involves a substantial shift in serum metabolic profiles

Kalhari Bandara Goonewardene[1,6], Naama Karu[2,6,ς], Khawaja Ashfaque Ahmed[1,6,*], Shelly Popowich[1], Betty Chow-Lockerbie[1], Lisanework E. Ayalew[1], Ruwani Karunarathna[1], Thushari Gunawardana[1], Mengying Liu[1], Suresh K. Tikoo[3], Marianna Foldvari[4], Philip Willson[5], Rupasri Mandal[2], David S. Wishart[2] and Susantha Gomis[1,*]

[1]Department of Veterinary Pathology, Western College of Veterinary Medicine, University of Saskatchewan, Saskatoon SK, S7N 5B4, Canada

[2]Department of Biological Sciences and Computing Science, University of Alberta, Edmonton, AB, T6G 2E9, Canada.

[3] Vaccinology and Immunotherapy, School of Public Health, University of Saskatchewan, Saskatoon, SK, S7N 5E3, Canada.

[4] School of Pharmacy, University of Waterloo, 200 University Avenue West, Waterloo, ON, N2L 3G1, Canada.

[5] Canadian Centre for Health and Safety in Agriculture, University of Saskatchewan, Saskatoon, Canada, SK, S7N 5E5, Canada.

*Corresponding authors mailing address: Department of Veterinary Pathology, Western College of Veterinary Medicine, 52 Campus Drive, University of Saskatchewan, Saskatoon, SK Canada S7N 5B4. Phone: (306) 966-7299. Fax: (306) 966-7439. Email: susantha.gomis@usask.ca (S.G); kaa201@mail.usask.ca (K.A.A)

[6] These authors contributed equally as co-first authors.

[ς] *Current address:* Analytical Biosciences and Metabolomics, Division of Systems Biomedicine and Pharmacology, Leiden Academic Centre for Drug Research, Leiden University, 2300RA Leiden, The Netherlands

Supplementary figure 1.

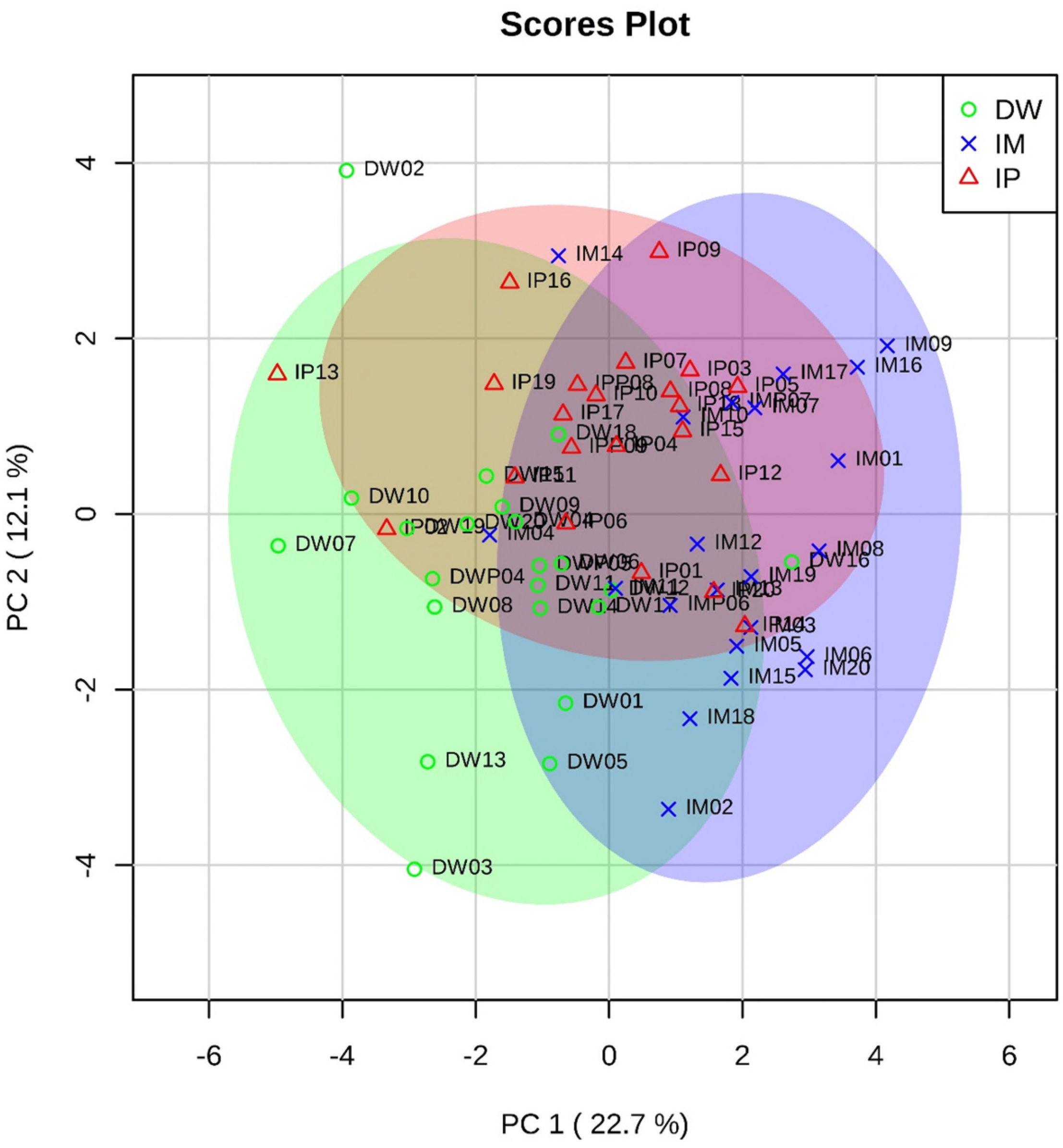

Figure S1. Scores plot of PCA obtained for 60 serum samples and 6 pooled samples (2 per experimental group), based on 40 metabolite concentrations. Data were log-transformed and autoscaled prior to analysis. DW, DW controls (green); IM, IM CpG-ODN (blue); IPL, IPL CpG-ODN (red).

Supplementary tables

Table S1. One-way ANOVA with Tukey's HSD Post-hoc comparison between metabolite concentrations obtained for the experimental groups (DW, DW controls n=18; IM, IM CpG-ODN n=20; IPL, IPL CpG-ODN n=20).

Metabolite	F value	ANOVA P value	FDR – adjusted P value	Tukey's HSD significant pairs
Betaine	24.13	2.99E-08	1.20E-06	IM-DW; IPL-IM
Serine	16.29	2.77E-06	5.55E-05	IM-DW; IPL-IM
Tyrosine	14.79	7.24E-06	8.12E-05	IM-DW; IPL-IM
Proline	14.62	8.12E-06	8.12E-05	IM-DW; IPL-DW
Choline	13.22	2.05E-05	1.64E-04	IM-DW; IPL-DW
Alanine	10.44	1.44E-04	9.58E-04	IM-DW; IPL-IM
Hypoxanthine	9.67	2.51E-04	1.40E-03	IM-DW; IPL-DW
Glycine	9.53	2.80E-04	1.40E-03	IM-DW; IPL-DW
Cytidine	9.16	3.70E-04	1.64E-03	IM-DW; IPL-DW
Threonine	8.77	4.94E-04	1.98E-03	IM-DW; IPL-DW
Lysine	7.35	0.001	0.005	IM-DW; IPL-IM
Acetate	6.86	0.002	0.007	IM-DW; IPL-DW
2-Hydroxybutyrate	6.83	0.002	0.007	IM-DW; IPL-DW
Aspartate	6.51	0.003	0.008	IM-DW; IPL-DW
Glutamine	6.47	0.003	0.008	IM-DW; IPL-DW
Methionine	5.44	0.007	0.016	IM-DW; IPL-IM
Valine	5.40	0.007	0.016	IM-DW; IPL-IM
Creatine	5.39	0.007	0.016	IPL-IM
Isoleucine	4.96	0.011	0.022	IPL-IM
Uridine	4.61	0.014	0.027	IM-DW; IPL-DW
Citrate	4.60	0.014	0.027	IPL-DW; IPL-IM
D-Glucose	4.37	0.017	0.031	IM-DW
Formate	3.60	0.034	0.059	IPL-DW; IPL-IM
Fumarate	3.54	0.036	0.060	IPL-DW
Leucine	3.29	0.045	0.069	IM-DW; IPL-IM
Myo-inositol	3.28	0.045	0.069	IPL-DW
Tryptophan	2.92	0.062	0.092	IPL-DW
3-Hydroxybutyrate	2.71	0.075	0.108	IPL-DW

Table S2. Top pathway enrichment results for CpG-ODN treatments compared to DW controls.

Metabolic pathway	hits	impact	IPL CpG-ODN vs. DW control		IM CpG-ODN vs. DW control	
			P value	FDR-adjusted P value	P value	FDR-adjusted P value
Pyruvate metabolism	4/23	0.35	0.002	0.012	0.069	0.076
Citrate cycle	4/20	0.20	0.030	0.053	0.483	0.494
Glyoxylate and dicarboxylate metabolism	8/32	0.18	3.78E-04	0.005	2.00E-05	2.94E-04
Glycolysis or Gluconeogenesis	3/26	0.13	0.002	0.012	0.018	0.023
Glycine, serine and threonine metabolism	7/34	0.55	3.22E-04	0.006	1.19E-06	5.25E-05
Alanine, aspartate and glutamate metabolism	8/28	0.54	0.004	0.016	9.0E-4	0.003
Arginine and proline metabolism	4/38	0.18	0.009	0.030	9.66E-05	0.001
Arginine biosynthesis	4/13	0.07	9.05E-04	0.010	0.001	0.004
